# Regulator of G Protein Signaling 2 (RGS2) and RGS4 Form Distinct G Protein-Dependent Complexes with Protease Activated-Receptor 1 (PAR1) in Live Cells

**DOI:** 10.1371/journal.pone.0095355

**Published:** 2014-04-17

**Authors:** Sungho Ghil, Kelly L. McCoy, John R. Hepler

**Affiliations:** 1 Department of Life Science, Kyonggi University, Suwon, Republic of Korea; 2 Department of Pharmacology, Rollins Research center, Emory University School of Medicine, Atlanta, Georgia, United States of America; Medical School of Hannover, Germany

## Abstract

Protease-activated receptor 1 (PAR1) is a G-protein coupled receptor (GPCR) that is activated by natural proteases to regulate many physiological actions. We previously reported that PAR1 couples to G_i_, G_q_ and G_12_ to activate linked signaling pathways. Regulators of G protein signaling (RGS) proteins serve as GTPase activating proteins to inhibit GPCR/G protein signaling. Some RGS proteins interact directly with certain GPCRs to modulate their signals, though cellular mechanisms dictating selective RGS/GPCR coupling are poorly understood. Here, using bioluminescence resonance energy transfer (BRET), we tested whether RGS2 and RGS4 bind to PAR1 in live COS-7 cells to regulate PAR1/Gα-mediated signaling. We report that PAR1 selectively interacts with either RGS2 or RGS4 in a G protein-dependent manner. Very little BRET activity is observed between PAR1-Venus (PAR1-Ven) and either RGS2-Luciferase (RGS2-Luc) or RGS4-Luc in the absence of Gα. However, in the presence of specific Gα subunits, BRET activity was markedly enhanced between PAR1-RGS2 by Gα_q/11_, and PAR1-RGS4 by Gα_o_, but not by other Gα subunits. Gα_q/11_-YFP/RGS2-Luc BRET activity is promoted by PAR1 and is markedly enhanced by agonist (TFLLR) stimulation. However, PAR1-Ven/RGS-Luc BRET activity was blocked by a PAR1 mutant (R205A) that eliminates PAR1-G_q/11_ coupling. The purified intracellular third loop of PAR1 binds directly to purified His-RGS2 or His-RGS4. In cells, RGS2 and RGS4 inhibited PAR1/Gα-mediated calcium and MAPK/ERK signaling, respectively, but not RhoA signaling. Our findings indicate that RGS2 and RGS4 interact directly with PAR1 in Gα-dependent manner to modulate PAR1/Gα-mediated signaling, and highlight a cellular mechanism for selective GPCR/G protein/RGS coupling.

## Introduction

Extracellular signaling molecules such as neurotransmitters and hormones transmit their signals into cells by interacting with the large family (>900) of cell surface G protein coupled receptors (GPCRs) and linked heterotrimeric (Gαβγ subunits) GTP binding proteins (G proteins). The binding of extracellular signaling molecules to GPCRs activate G proteins by inducing the exchange of GDP for GTP on the Gα subunit. This facilitates Gα-GTP dissociation from the Gβγ dimer and release of G proteins from the receptor [1]. Dissociated Gα-GTP and Gβγ subunits interact with various downstream effectors and signaling pathways to mediate cell physiology. The life-time of this signaling event is dictated by the life-time of GTP bound to Gα, and is inactivated by the intrinsic GTPase activity characteristic of all Gα subunits. However, Gα GTPase activity is regulated and greatly accelerated by cofactor GTPase activating proteins (GAPs).

The most prominent GAPs for Gα subunits include the family (∼40 members) of regulators of G protein signaling (RGS) proteins, which bind to Gα-GTP and increase GTP hydrolysis [2]–[4]. RGS proteins are classified into several subfamilies based on their amino acid sequencing and protein structures [4], [5], and are characterized by a shared RGS domain (∼120 amino acid) that serves as the binding site for and confers the GAP activity onto Gα-GTP. Recent studies have suggested that RGS proteins also can interact directly or indirectly with GPCRs [6]–[9]. RGS proteins interact with GPCR via the receptor third intracellular loops (i3), C-termini, or by recruiting adapter proteins to modulate the functions of coupled G proteins [9], [10]. Therefore, compelling evidence indicates that GPCRs can selectively form a physical and functional complex with certain RGS protein and G proteins.

RGS proteins of the R4/B subfamily, including RGS1-5, 8, 13, 16, 18 and 21, are the smallest RGS proteins in size, each containing a single RGS domain with relatively small N and C-termini (except for RGS3) [5]. RGS2 is broadly expressed in both mouse and human tissues, and interacts with the G_q/11_ family to inhibit G_q/11_-mediated signaling [11]–[13]. RGS2 also associates with several types of adenylyl cyclase and regulates intracellular cAMP concentration [14]. RGS4 is mainly expressed in brain and cardiac tissues [15], [16], and interacts with Gα_i/o_ and Gα_q_ [11], [17], [18]. Previously, we reported that RGS2 and RGS4 can bind directly and selectively to GPCRs, to regulate the function of linked G proteins. These interactions are regulated by specific regions with the N-termini of the RGS proteins and the i3 loops of the GPCRs [6], [7].

Protease-activated receptors (PARs) are GPCRs that can be activated by proteases such as thrombin, trypsin and plasmin. Most GPCRs are activated by small hydrophilic molecules. By contrast, PARs are stimulated by proteolytic cleavage and binding of an intrinsic N-terminal ligand, which is cleaved by one or more endogenous proteases [19]. There exist four types of PARs termed PAR1-PAR4 in the order of their discovery. PAR1 was originally identified as a receptor for thrombin [20], and its functions have been widely studied in the cardiovascular and central nervous systems [21], [22]. In our previous study, PAR1 was shown to form stable complexes with various G proteins including members of the G_i_, G_q_ and G_12_ subfamilies. Stimulated PAR1 coupling to these G proteins induces activation of MAPK/ERK and inhibition of adenylyl cyclase (G_i/o_), activation of inositol phosphate accumulation and calcium release (G_q/11_), and activation of Rho activation (G_12/13_ and G_q/11_); PAR1 also stimulates migration of Neu7 glial cells [23], [24].

A large body of evidence now exists to suggest that GPCRs and RGS proteins can form a physical and functional complex to regulate G protein signaling [9], [10]. However what cellular factors dictate selective RGS/GPCR coupling is poorly understood. Most previous studies have used biochemical affinity pull-down assays to determine if RGS proteins can form a complex with GPCRs and/or G proteins. However, biochemical interactions such as these that depend on detergent extraction of receptors may not fully or accurately reflect functional GPCR/G protein/RGS protein complex formation in live cells. In addition, these studies have complicated experimental procedures. In the present study, we employed a bioluminescence resonance energy transfer (BRET) assay to investigate the mechanism whereby PAR1 and RGS proteins (RGS2 and RGS4) interact in live cells, in the presence or absence of G proteins. Here we report that PAR1 functionally interacts with both RGS2 and RGS4 in live cells, but only in a strict G protein-dependent manner to regulate PAR1/G protein signaling. Our findings suggest that both RGS2 and RGS4 each selectively modulate PAR1/Gα-mediated signaling by binding to PAR1 in a Gα-dependent manner.

## Materials and Methods

### Construction of Plasmids

The mouse PAR1 cDNA (GenBank accession number NM_010169) in pBSK was a generous gift from Dr. Stephen Traynelis (Emory University). pcDNA3.1-GαqYFP and –GαsYFP plasmids were kindly provided by Dr. Joe Blumer (Medical University of South Carolina). pcDNA3.1-Gαq, Gα_11_EE, -Gα_i_, -Gα_oA_EE, -Gα_12_EE, -Gα_13_EE and –Gα_s_EE plasmids were obtained from Missouri S&T cDNA Resource Center. To generate PAR1-Ven plasmid (Venus-N1-PAR1, C-terminal Venus tagged plasmid of PAR1), PAR1 cDNA was amplified from pBSK-PAR1 by PCR with the primers 5′-ATC GAT AAG CTT GAT ATC GAA TTC-3′ (forward), and 5′-TTT GGT ACC GCT AAT AGC TTT T-3′ (reverse). The amplified products were inserted into the Venus-N1 using *HindIII* and *KpnI* restriction enzymes. Oligonucleotide primers used to create RGS2-Luc plasmid (pRLuc-N3-RGS2, C-terminal luciferase tagged plasmid of RGS2HA) are as follows: 5′-CTT GGT ACC ACC ATG CAA AGT GCT ATG TTC −3′ (forward) and 5′-TTG GGC CCG AGC GTA ATC TGG AA-3′ (reverse). pcDNA3.1-RGS2-HA was used as a template, and amplified PCR products were inserted into pRLuc-N3, using *KpnI* and *ApaI* restriction enzymes. RGS4-HA-Luc plasmid (pRLuc-N3-RGS4, C-terminal luciferase tagged plasmid of RGS4-HA) were generated by PCR reaction with the primers, 5′-TTT AAA CTT AAG CTT GGT ACC ACC ATG TGC-3′ (forward) and 5′-TTG GGC CCG AGC GTA ATC TGG AA-3′ (reverse). pcDNA3.1-RGS4-HA was used as a template and amplified PCR products were inserted into pRLuc-N3 using *KpnI* and *ApaI* restriction enzymes. To make PAR1-FLAG plasmid (pcDNA3.1-PAR1-FLAG, C-terminal FLAG tagged plasmid of PAR1), PAR1 cDNA was amplified from pBSK-PAR1 by PCR with the primers, 5′-GAC GGT ATC GAT AAG CTT GAT ATC GAA TTC CCG GG-3′ (forward) and 5′-AAA CTC GAG CTA CTT GTC ATC GTC GTC CTT GTA GTC AGC TAA TAG CTT T-3′ (reverse). The amplified products were inserted into the pcDNA3.1 using *HindIII* and *XhoI* restriction enzymes. To generate a G_q/11_-insensitive mutant of PAR1 (R205A) [24] for BRET studies, the wild type mPAR1-Ven plasmid (above) was used as a template for amplification by QuickChange (Qiagen) using primers, 5′-CAT AAG CAT TGA CGC GTT CCT GGC GGT G-3′ (forward) and 5′-CAC CGC CAG GAA CGC GTC AAT GCT TAT G- 3′(reverse). The amplified product was then digested with *DpnII* to remove the parent DNA, transformed into bacterial strain XL-Blue, and DNA recovered from resulting colonies was sequenced to confirm insertion of mutant R205A.

### Cell Culture and Transfection

The COS7 cell line (American Type Culture Collection, ATCC, CRL 1651) was maintained in DMEM (without phenol red) supplemented with 10% fetal bovine serum, 2 mM glutamine, 100 units/ml penicillin, and 100 µg/ml streptomycin. Cells were incubated at 37°C with 5% CO_2_ in a humidified incubator. Cells were transiently transfected with the indicated plasmids using lipofectamine 2000, following the manufacturer’s instructions.

### BRET Assays

BRET assays were performed in the same manner as has been previously described [25], [26]. Briefly, COS7 cells were transfected with BRET donor (luciferase-tagged) and acceptor (Venus-tagged) plasmids. After 48 h, cells were washed with PBS and harvested with Tyrode’s solution (140 mM NaCl, 5 mM KCl, 1 mM MgCl_2_, 1 mM CaCl_2_, 0.37 mM NaH_2_PO_4_, 24 mM NaHCO_3_, 10 mM HEPES, and 0.1% glucose, pH 7.4). Cells were distributed in triplicate at 1×10^5^ cells/well into gray 96-well OptiPlates (PerkinElmer Life Science). Venus-tagged protein expression levels were measured by using TriStar LB941 plate reader (Berthold Technologies) with excitation and emission filters at 485 nm and 535 nm, respectively. To measure BRET signals, the cells were treated with luciferase substrate, coelenterazine H (Nanolight Technology, final concentration 5 µM). After 2 min, the luminescence was measured by using 480±20 and 530±20 nm filters. BRET signals were determined by calculating the ratio of the light intensity emitted by the Venus divided by the light intensity emitted by luciferase. Net BRET values were corrected by subtracting the background BRET signal detected from expression of the Luciferase alone. The expression levels of Gα protein were determined by immunoblot, using antibodies against Gα_q_, Gα_i1_, Gα_o_, Gα_12_ (all Gα antibodies from Santa Cruz Biotechnology) and EE-epitope tag (Covance).

### Generation and Purification of GST-PAR1 Intracellular Loop Fusion Proteins

cDNA encoding glutathione-S-transferase (GST)-PAR1-i2, GST-PAR1-i3, and GST-PAR1-i3 were cloned into the pGEX4T vector. For this, the second intracellular loop (i2 loop) or the i3 loop of PAR1 was each amplified by PCR from corresponding regions of the mouse full-length receptor including *EcoRI* and *XhoI* cut site as linkers. These fragments were cloned in frame of the pGEX4T vector (*EcoRI/XhoI*) encoding an N-terminal GST tag.

For protein production, plasmid constructs encoding GST-PAR1 i2 and i3 loop fusion proteins were transformed into *E. coli* strain BL21/DE3 for 2 h at 37°C with shaking. Cells were centrifuged, and pellets were frozen at −80°C. Pellets were thawed in harvest buffer (10 mM HEPES pH 8, 50 mM NaCl, 5 mM EDTA, 0.5% Triton-X100) supplemented with protease inhibitors and lysozyme, sonicated, and then centrifuged at 4°C to yield bacterial cell lysates. Lysates were combined and mixed with Glutathione-sepharose 4B beads (Amersham-Pharmacia) for 1 h, at 4°C. Protein-bead complexes were recovered and then washed with harvest buffer, and stored as slurry solutions in harvest buffer at −80°C until experimentation. The protein concentration present in the slurries was determined by Coomassie blue staining versus BSA standards, and the same amount of total protein was used for in each binding reaction.

### RGS Affinity Pull-down Assays

RGS protein affinity pull-down assays were performed as previously described [6], [7]. Briefly, Glutathione-sepharose 4B beads containing equal amounts of GST-PAR1-i2 or GST-PAR1-i3 fusion proteins (above) were mixed with equal amounts of purified His-tagged RGS proteins in a total reaction volume of 250 µL of reaction buffer (30 mM Imidazole and 80 mM NaCl, 10 mM HEPES, pH 7.4 final to minimize non-specific binding), and reactions were mixed overnight at 4°C. Beads were pelleted by centrifugation and were washed with harvest buffer. Proteins bound to the beads were eluted with 2X sample buffer and were detected by immunoblot.

### Measurement of Erk Activation in Cells

COS-7 cells were transiently transfected with empty vector (pcDNA3.1), vector encoding PAR1 alone, or PAR1 together with vector encoding C-terminally HA-tagged RGS2 (RGS2-HA), RGS4-HA, or RGS16-HA. After overnight serum starvation, cells were stimulated with 20% serum (positive control) or with PAR1 peptide agonist (30 µM TFLLR) for 2–5 min, harvested, sonicated, boiled in sample buffer, subjected to Western blot analysis with p44/42 ERK1/2 and phospho-p44/42 ERK1/2 antibodies (Cell Signaling Technology) at 4°C. Detection of the HA-tagged RGS proteins was performed by immunoblotting the same samples used in the ERK1/2 phosphorylation experiments.

### Measurement of Calcium Signaling in *Xenopus Laevis* Oocytes by Two-electrode Voltage Clamp Recordings

Oocytes were harvested from *X. laevis* were defolliculated and maintained in 1x Barth’s culture solution at 16°C. Stage V–VI oocytes were either injected with 5 ng PAR1 cRNA, which was synthesized from cDNA according to the manufacturer’s specifications (Ambion). Recordings were performed 4–5 days after injections. The recording solution contained 60 mM NaCl, 38 mM KCl, 2.3 mM CaCl_2_, 1 mM MgCl_2_, and 6 mM HEPES (pH 7.4). Patch pipettes with tip diameters of 1–2 µm were used as electrodes and filled with 300 mM KCl. Current responses were recorded at a holding potential of −40 mV. Data was acquired and voltage was controlled with a two-electrode voltage-clamp amplifier (OC-725; Warner Instruments). PAR1-agonist peptide TFLLR was diluted in 1x Barth’s to final concentrations of 30 µM and was used to elicit the ICl (Ca). For studies with RGS protein regulation of PAR1 signaling, 5 ng cRNA for each RGS was added with 5ng PAR1 cRNA prior to injection. Recordings were performed 4–5 days after injections as detailed above.

### Measurement of RhoA Activation

The GTP-bound form of RhoA was measured using the absorbance-based RhoA Activation G-LISA kit (Cytoskeleton Inc.) according to the manufacturer’s protocol. COS-7 cells were transiently transfected with cDNA encoding empty vector (control) or with vector encoding C-terminally HA-tagged RGS2 (RGS2-HA), RGS4-HA or RGS16-HA. Prior to assay, transfected COS-7 cells were serum-starved overnight and then treated for 2 min with TFLLR. The absorbance from the G-LISA plate was read by a spectrophotometer at a wavelength of 490 nm.

## Results

### 1. Interaction between PAR1 and RGS2 in Live Cells

**Figure 1:**
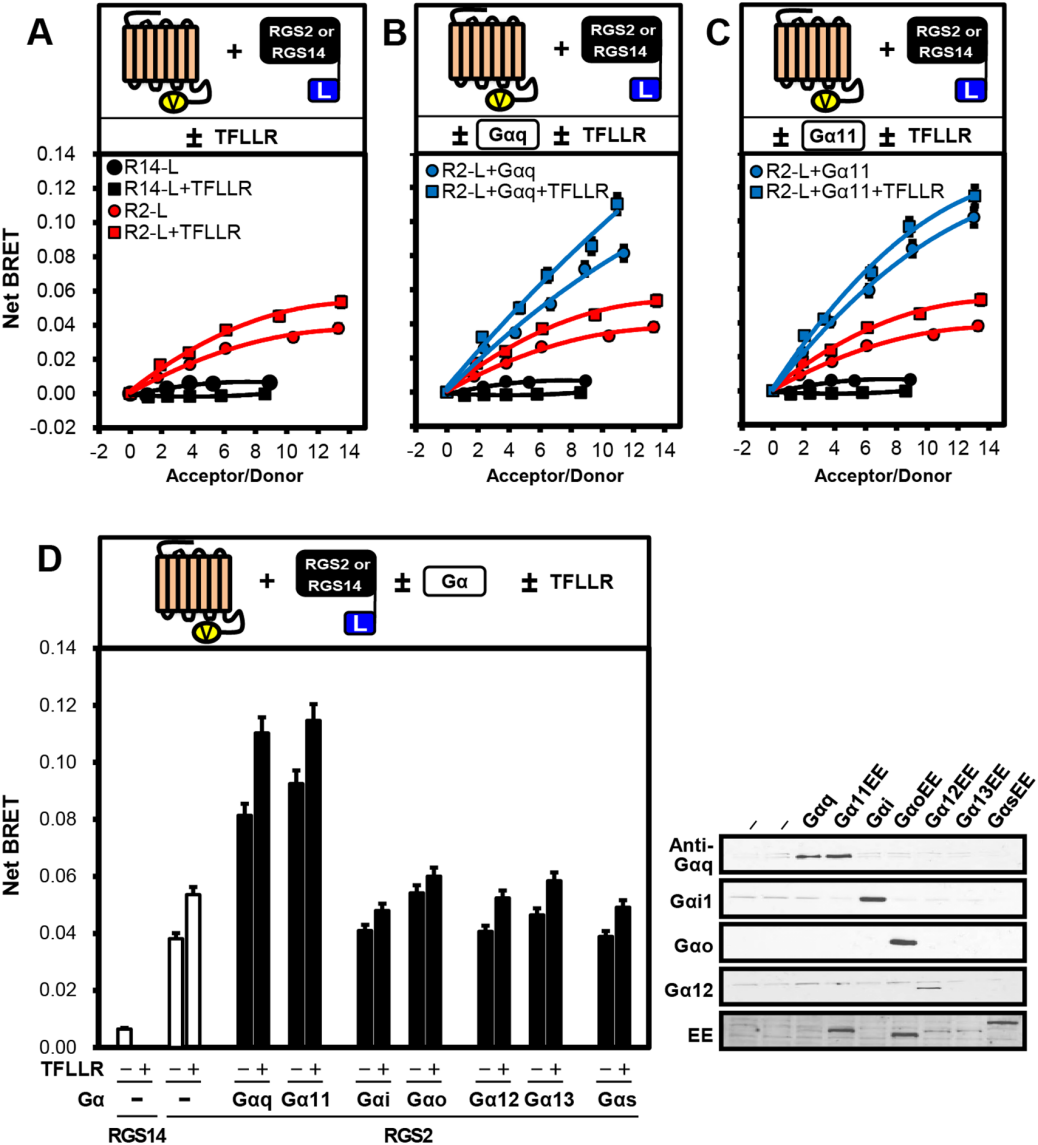
RGS2 interacts with PAR1 in live cells. **A**, Top panel: Cartoon illustrating proteins and conditions used in the experiment. Bottom panel: COS7 cells transfected with an increased amount of PAR1-Ven (0, 0.25, 0.5, 1.0, 1.5, 2.0 µg), together with a fixed amount of RGS2-Luc (35 ng) or RGS14-Luc (5 ng), were subjected to the BRET assay in both the absence and presence of 30 µM of TFLLR. Net BRET signals are shown between PAR1-Ven and either RGS2-Luc or RGS14-Luc. **B** and **C**, Top panel: Cartoons illustrating proteins and conditions used in the experiment. Bottom panel: COS7 cells transfected with an increased amount of PAR1-Ven, together with fixed amount of RGS2-Luc or RGS14-Luc, were subjected to the BRET assay in both the absence and presence of untagged Gα_q_ (B), Gα_11_ (C) and TFLLR. Bottom panel, net BRET signals are shown between PAR1-Ven and either RGS2-Luc or RGS14-Luc. The black and red plots in (B) and (C) were identical to those in (A). **D**, Top panel: Cartoon illustrating proteins and conditions used in the experiment. Bottom panel: COS7 cells were transfected with both fixed amount of PAR1-Ven (2.0 µg) and either RGS2-Luc (35 ng) or RGS14-Luc (5 ng), and the cells were subjected to the BRET assay in both the absence and presence of 0.5 µg of untagged Gα and TFLLR. Bottom panel, net BRET signals are shown between PAR1-Ven and either RGS2-Luc or RGS14-Luc. Right panel, shows a representative immunoblot of the different untagged Gα subunits used in the BRET experiment. All BRET graphs are representative of at least three independent experiments. Abbreviations: R14-L, RGS14-Luc; R2-L, RGS2-Luc.

Previous reports indicate that some RGS proteins interact directly with GPCRs to modulate their signals [9], [10]. To determine whether RGS2 interacts with PAR1 in live cells, we performed BRET assays using PAR1-Venus (PAR1-Ven) and RGS2-Luciferase (RGS2-Luc) plasmids. As a negative control, we used RGS14-Luc, which we recently reported interacts with the α2-adrenergic receptors in cells using BRET assays [25], [26]. In COS7 cells transfected with PAR1-Ven and a fixed amount of RGS2-Luc as donor (35 ng), the BRET signals increased with increasing amounts of PAR1 acceptor, whereas no BRET signals were detected between PAR1-Ven and RGS14-Luc (Fig. 1A). In the presence of the PAR1 peptide agonist TFLLR, the BRET signals between PAR1-Ven and RGS2-Luc were increased compared with no agonist. RGS2 is coupled with G_q/11_ family, and inhibits their functions [11], [12]. To investigate the dependency of PAR1·RGS2 interaction on the presence of G_q/11_, we co-transfected cells with untagged Gα_q_ or Gα_11_ together with PAR1-Ven and RGS2-Luc. Expression of Gα_q_ or Gα_11_ significantly increased the interaction between acceptor and donor, and this signal was marginally enhanced by the addition of TFLLR (Fig. 1B and C). We next tested the effect of other Gα subunits on PAR1·RGS2 interaction and observed that the BRET signals were not altered in the presence of Gα_i/o_, Gα_12/13_ or Gα_s_ (Fig. 1D and Fig. S1). Taken together, these results indicate that RGS2 can interact with PAR1 in live cells, and that their interaction is markedly promoted by the presence of either Gα_q_ or Gα_11_ and by receptor agonist, but not by other Ga subunits.

**Figure 2:**
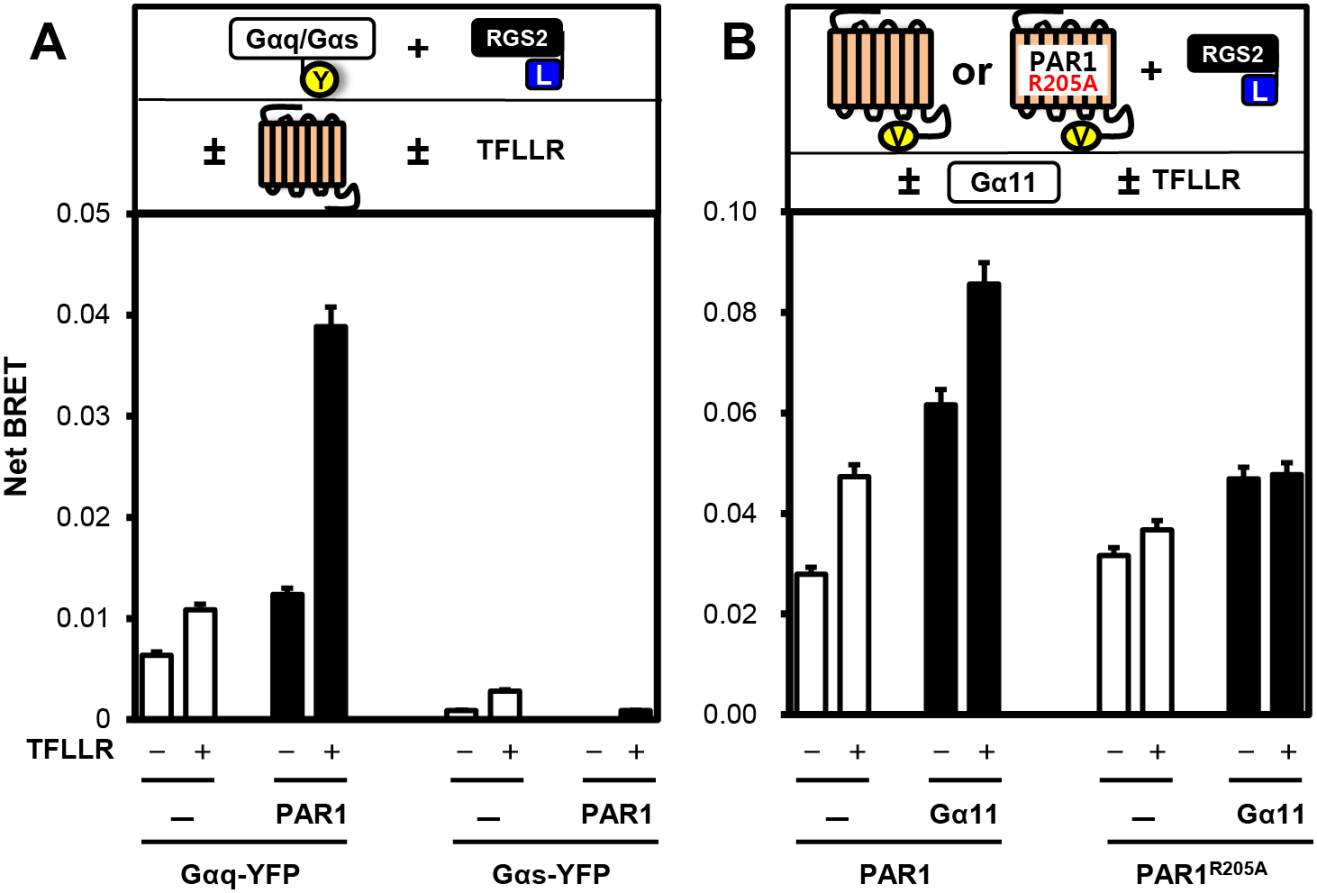
PAR1 forms a complex with RGS2 and Gα_q/11_. **A**, Top panel: Cartoon illustrating proteins and conditions used in the experiment. Bottom panel: COS7 cells transfected with RGS2-Luc (35 ng) and either Gα_q_-YFP or Gα_s_-YFP (0.75 µg) were subjected to the BRET assay in both the absence and presence of 0.5 µg of untagged PAR1-FLAG and 30 µM of TFLLR. Net BRET signals are shown between RGS2-Luc and either Gα_q_-YFP or Gα_s_-YFP. **B**, Top panel, Cartoon illustrating proteins and conditions used in the experiment. Bottom panel: COS7 cells transfected with RGS2-Luc (35 ng) and either PAR1-Ven or PAR1^R205A^-Ven (1.5 µg) were subjected to the BRET assay in both the absence and presence of 0.5 µg of untagged Gα_11_ and TFLLR. Net BRET signals are shown between RGS-Luc and either PAR1-Ven or PAR1^R205A^-Ven.

To further explore if receptor/Gα_q_ coupling promotes interaction between PAR1 and RGS2, we co-transfected Gα_q_-YFP, RGS2-Luc and/or PAR1-FLAG, and the cells were subjected to the BRET assay. Gα_s_-YFP was used as a negative control. As before, BRET signals between Gα_q_-YFP and RGS2-Luc were increased by the expression of PAR1-FLAG (Fig. 2A) and further increased by the PAR1 agonist TFLLR. By contrast, no BRET signals were observed with Gα_s_-YFP (Fig. 2A). We previously reported that mutation of a single arginine residue of PAR1 (PAR1^R205A^) disrupted binding to and functional coupling with Gα_q/11_ [24]. We investigated the effects of PAR1^R205A^ on complex formations with RGS2 and Gα_11_. Whereas the PAR1^R205A^ mutant can still interact weakly with RGS2 similar to wild type PAR1, this binding was not further enhanced by Gα_11_ (Fig. 2B), indicating that the PAR1/Gα_q/11_ complex is a preferred substrate for RGS2 in live cells.

### 2. Interaction between PAR1 and RGS4 in Live Cells

**Figure 3:**
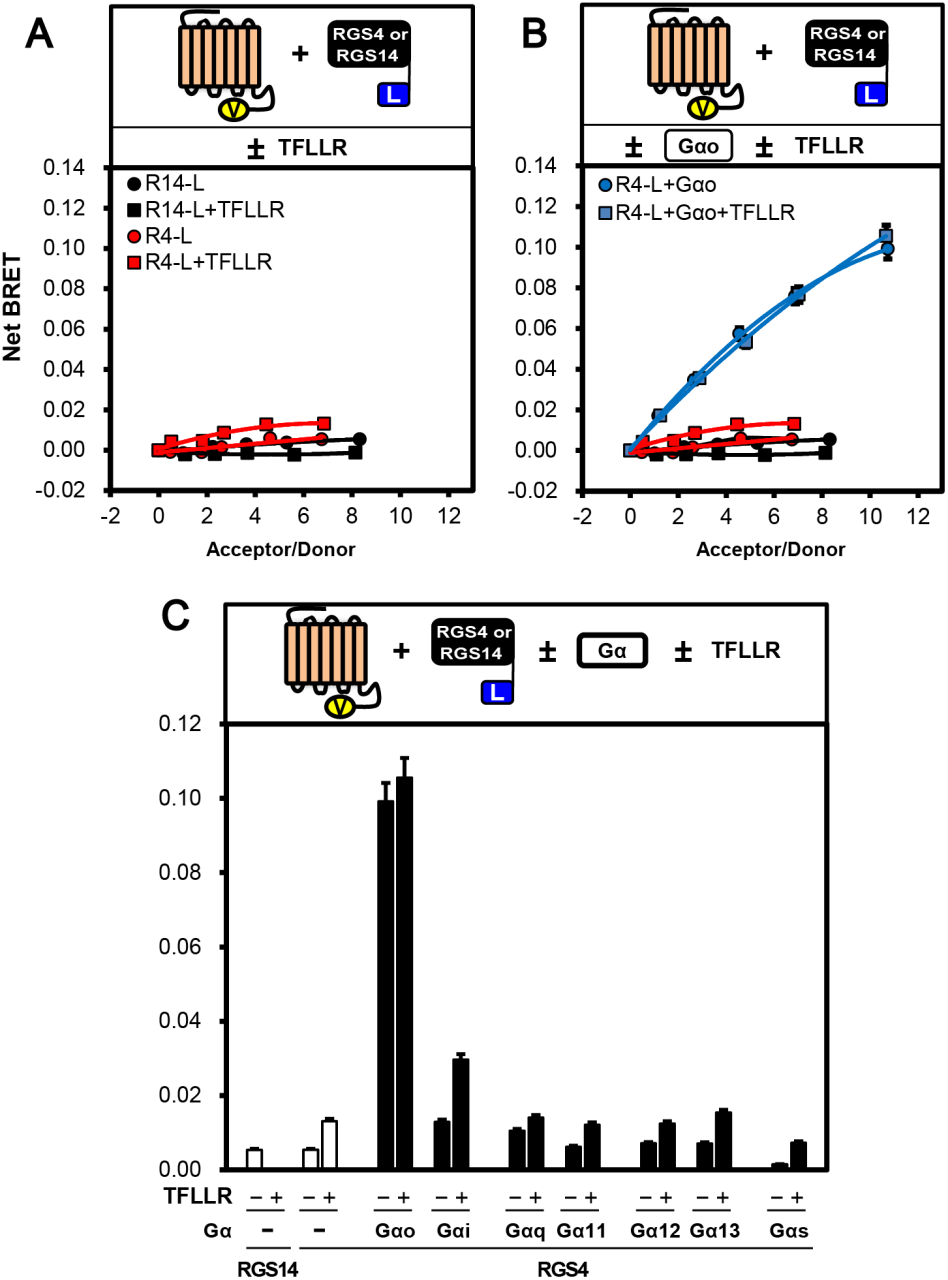
RGS4 interacts with a PAR1/Gα_o_ complex in live cells. **A**, Top panel: Cartoon illustrating proteins and conditions used in the experiment. Bottom panel: COS7 cells were transfected with increasing amounts of PAR1-Ven (0, 0.1, 0.25, 0.5, 1.0, 1.5 µg), together with fixed amount of RGS4-Luc (45 ng) (Red symbols) or RGS14-Luc (5 ng) (Black symbols), and the cells were subjected to BRET analysis in both the absence and presence of 30 µM of TFLLR. Net BRET signals are shown between PAR1-Ven and either RGS4-Luc or RGS14-Luc. **B**, Top panel: Cartoon illustrating proteins and conditions used in the experiment. Bottom panel: COS7 cells were transfected with an increasing amount of PAR1-Ven together with fixed amount of RGS4-Luc (Red symbols) or RGS14-Luc (Black symbols) as in A, and were subjected to BRET analysis in both the absence or presence of untagged Gα_o_ (Blue symbols) and TFLLR. Net BRET signals are shown between PAR1-Ven and either RGS4-Luc or RGS14-Luc. (NOTE: The black and red plots in (B) were identical to those in (A)). **C,** Top panel: Cartoon illustrating proteins and conditions used in the experiment. Bottom panel: COS7 cells were transfected with both fixed amount of PAR1-Ven (1.5 µg) and either RGS4-Luc (45 ng) or RGS14-Luc (5 ng), and the cells were subjected to the BRET assay in both the absence and presence of 0.5 µg of untagged Gα and TFLLR. Net BRET signals are shown between PAR1-Ven and either RGS4-Luc or RGS14-Luc. Abbreviations used are R14-L  =  RGS14-Luc; R4-L  =  RGS4-Luc.

Like RGS2, RGS4 binds directly to GPCRs, including opioid receptors, and modulates their functions [8]. Therefore, to investigate whether RGS4 binds to PAR1 here in live cells, we co-transfected with PAR1-Ven and RGS4-Luc, and the cells were subjected to the BRET assay. Unlike RGS2 (Fig. 1A), RGS4 had weak binding properties to PAR1 in the absence of Gα in live cells (Fig. 3A). However, high levels of BRET activity were observed only in the presence of Gα_o_ (Fig. 3B and C), with lower signals evident with Gα_i_, and even less BRET activity with Gα_q_, Gα_11_, Gα_12_, Gα_13_ and Gα_s_ (Fig. 3C and Fig. S2), suggesting that PAR1 binding to RGS4 may be Gα_o_-dependent.

### 3. RGS2 and RGS4 Bind Directly, and Selectively to the i3 Loop of PAR1

**Figure 4:**
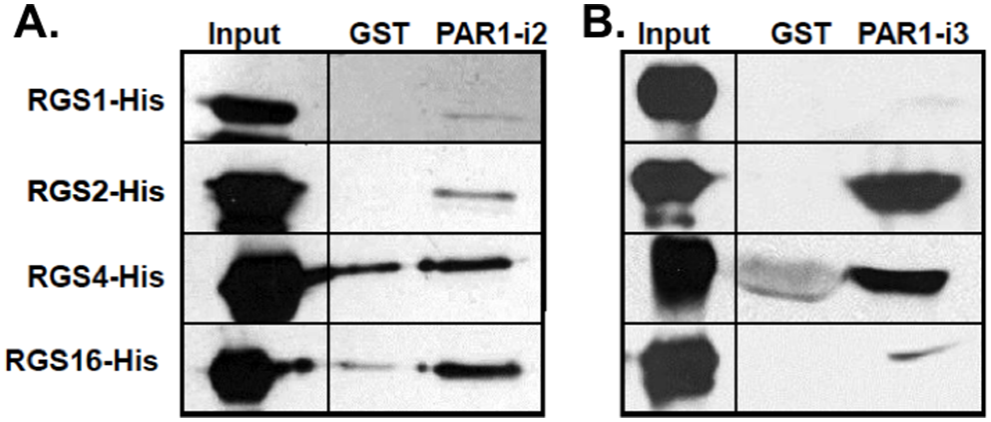
RGS2 and RGS4 bind directly and selectively to the i3 loop of PAR1. Purified RGS1-His, RGS2-His, RGS4-His or RGS16-His were incubated with equal amounts of GST alone, GST-PAR1-i2, or with GST-PAR1-i3 bound to glutathione-Sepharose beads. After centrifugation, bound RGS proteins were eluted in 2X sample buffer and subjected to SDS-PAGE. Immunoblots were performed using an anti-His antibody.

To determine whether PAR1 binds directly to the RGS2 and RGS4, and consequently which domain of PAR1 might be the main contributor to binding of the RGS proteins, we purified His-tagged RGS proteins (RGS1-His, RGS2-His, RGS4-His and RGS16-His), and GST-tagged PAR1-i2 and -i3 domains from a bacterial culture, and performed a GST-affinity pull-down assays. RGS2 and RGS4, but not RGS1 or RGS16 bound robustly to the PAR1 i3 loop but not GST. By comparison, RGS2, RGS4 and RGS16 bound only weakly to the i2 loop (compared to GST alone) and RGS1 did not bind to either PAR1 i2 or i3 (Fig. 4A and B).

### 4. Effect of RGS2 and RGS4 on PAR1-Gα-Mediated Signaling

**Figure 5:**
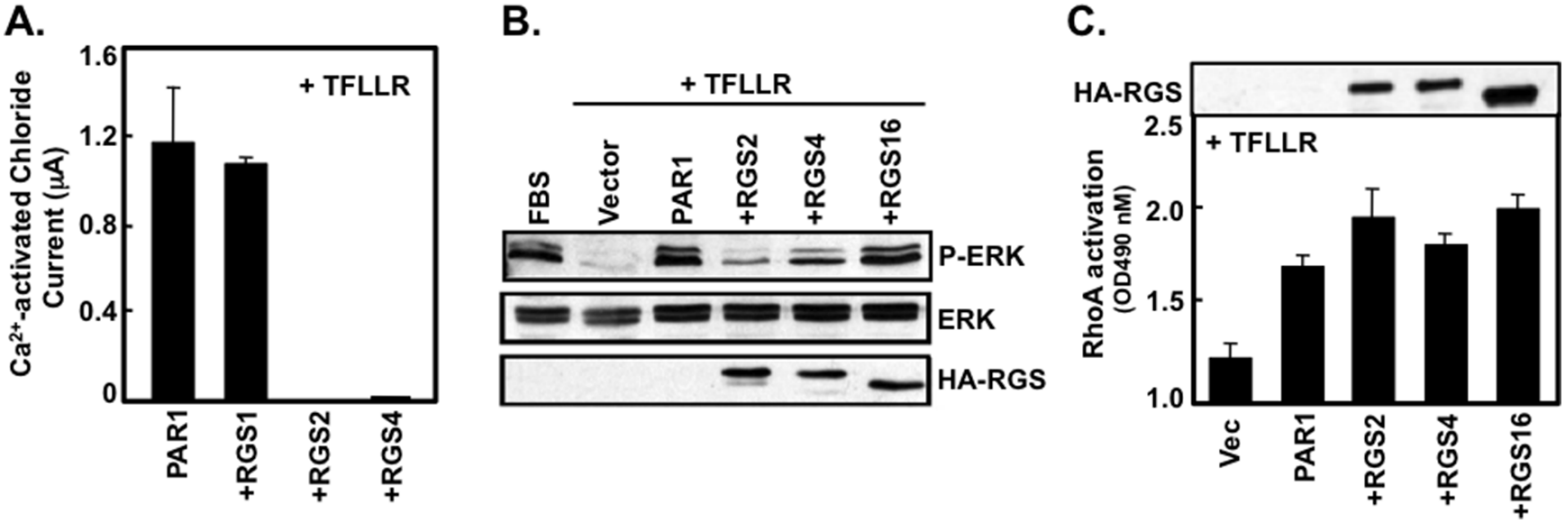
RGS2 and RGS4 selectively inhibit PAR1/Gα-mediated signaling in live cells. **A**, RGS2 and RGS4, but not RGS1, reduce PAR1-evoked calcium activated chloride currents in oocytes. PAR1 cRNA alone or mixed with individual RGS protein cRNA was injected into *X. laevis* oocytes, which were sustained in 1x Barth’s solution. 4–5 days after injection, Ica (Cl) measurements were obtained from the oocytes in response to activation with 30 mM TFLLR. A two electrode voltage clamp was used to obtain the current changes, as described in Materials and Methods. Data were entered into a Microsoft Excell spreadsheet which was used to calculate the mean change in Ica (Cl) + S.E.M. (n≥11 oocytes). **B**, RGS2 and RGS4 differentially block PAR1-stimulated ERK1/2 phosphorylation. Vector alone or PAR1 alone, or pairs of PAR1 and the indicated RGS protein were separately transfected into COS-7 cells. Cells were either stimulated with 20% serum or 30 µM TFLLR for 5 min. Immunoblots were performed with either phospho-ERK1/2, total ERK1/2, or an anti-HA antibody, followed by a goat-anti rabbit secondary antibody and detected by ECL. **C**, PAR1-mediated RhoA activation is not regulated by RGS proteins. As described in the materials and methods section, RhoA activation was measured using a RhoA G-LISA Assay kit. Vector alone, PAR1 alone, or PAR/RGS pairs were separately transfected into COS-7 cells for 5 h before an overnight period of serum starvation. The next day, cells were stimulated with 30 µM TFLLR for 2 min prior to cell lysis. The manufacturer’s protocol was followed throughout the experiment, and the absorbance of each well was read with a spectrophotometer wavelength of 490 nm. Data were entered into a Microsoft Excel spreadsheet which was used to calculate the mean fold change in absorbance (bars) over basal levels plus the S.E.M (error bars), n = 3 for each condition.

PAR1 relies upon Gα_q/11_ to activate PLC-β and linked intracellular InsP_3_-calcium signaling [23], [24]. Therefore, to determine whether RGS2 and RGS4 inhibit PAR1-directed Gα_q/11_ signaling, we measured the capacity of RGS2 and RGS4 to modulate calcium-activated chloride channels in *Xenopus* oocytes. Our previous studies showed that RGS4 blocks a chloride channel current in *Xenopus* oocytes that is activated by a GPCR/G_q/11_/Ins (1,45) P_3_/calcium pathway, and that RGS4 acts by blocking G_q_/calcium signaling but does not directly affect chloride channel function [27]. In the presence of TFLLR, PAR1 activated the calcium-dependent chloride channel and expression of either RGS2 or RGS4 but not RGS1 each completely blocked this PAR1-Gα_q/11_-stimulated chloride current (Fig. 5A), indicating that RGS2 and RGS4 inhibit PAR1-Gα_q/11_-mediated calcium signaling. We next tested if RGS2 or RGS4 regulates PAR1-Gα stimulated MAPK/ERK signaling. GPCR activation generally stimulates MAPK/ERK signaling through multiple G protein-mediated signaling cascades [28]. In our previous study, PAR1 activation was shown to induce MAPK/ERK phosphorylation, and this effect is completely blocked by PTX, indicating PAR1 can stimulate MAPK/ERK signaling in a G_i/o_-dependent manner [23]. To investigate whether RGS2 and RGS4 inhibit MAPK/ERK phosphorylation, the MAPK/ERK phosphorylation assay was performed by using phospho-ERK antibodies. We transfected PAR1 and either HA-tagged RGS2, RGS4 or RGS16 in COS7 cells, and activated the receptors using TFLLR. Fig. 5B shows that expression of either RGS2 or RGS4 inhibits MAPK/ERK phosphorylation to some extent, whereas no effect was observed with RGS16 indicating that MAPK/ERK signaling induced by PAR1/G protein is limited by RGS2 and RGS4.

Signaling through the G_12/13_ family transduces GPCR signals into RhoA activation, actin remodeling, and assembly of focal adhesions [29], [30]. In addition, PAR1 uses G_12/13_ family to activate RhoA as we have previously reported [23]. To elucidate whether RGS2 and RGS4 inhibit PAR1/G_12_-mediated Rho signaling, we performed Rho activity assay in COS7 cells transfected with PAR1 alone, or together with HA-tagged RGS2, RGS4 or RGS16. Even when RGS proteins were highly expressed in the cells, RhoA activation was not altered compared to PAR1 alone, indicating that RGS2 and RGS4 do not affect PAR1/G_12/13_-mediated RhoA signaling (Fig. 5C). Taken together, these data suggest that RGS2 and RGS4 selectively inhibit PAR1/Gα-mediated signaling.

## Discussion

In this study, we investigated whether RGS2 and RGS4 interact with PAR1 receptors in live cells, and we also determined whether their interaction affected PAR1/Gα-mediated signaling. Our findings (Fig. 1–4) show that RGS2 and RGS4 interacted with PAR1 in live cells in a Gα-dependent manner, and that these interactions may be direct. RGS2 selectively formed a complex with PAR1 and G_q/11_, whereas RGS4 selectively formed a complex with PAR1 in the presence of Gα_o_. Figure 5 shows that PAR1-Gα-mediated calcium and MAPK/ERK signaling was inhibited by RGS2 and RGS4. Although PAR1 couples with G_12/13_ family and increases RhoA activity in the presence of TFLLR [23], RGS2 and RGS4 did not alter the PAR1/G_12_-mediated RhoA activation in the presence of TFLLR. This may be due to the fact RGS2 and RGS4 preferentially interact with G_q_ and G_i_/G_q_ family members, respectively [11], [12].

It is well established that RGS2 selectively binds to Gα_q/11_ to inhibit their signaling [11]. However, low levels of BRET signals between G_q_-YFP and RGS2-Luc were displayed in the absence of PAR1 and TFLLR (Fig. 2A). The RGS homology domain of RGS proteins interacts with the “switch” region located within the alpha helical domains of Gα [31], [32]. The coding sequence of YFP in Gα_q_-YFP was inserted in the alpha helical domain (αb-αc loop) of Gqα [33], [34]. We consider the possibility that insertion of YFP in Gα_q_ might cause a change of Gα_q_ protein conformation, so as to decrease affinity with RGS2. However, the BRET signals between G_q_-YFP and RGS2-Luc were increased in the presence of untagged PAR1 and TFLLR (Fig. 2A), indicating that activation of PAR1 can induce conformational change of both proteins and, as a result, induce formation of a PAR1·Gα_q_ -YFP·RGS2-Luc protein complex.

GPCRs are integral membrane proteins that possess seven-transmembrane domains. They contain three extracellular loops and three (or in some cases four) intracellular loops. Intracellular loops contribute to direct interaction with various signaling and regulatory proteins including many of G proteins, RGS proteins, arresins, kinases, cytoskeletal-associated proteins, and other GPCRs [35]. The second intracellular (i2) loop of PAR1 contributes to Gα coupling [24], [36]. The Arg-205 residue in the i2 loop of PAR1 plays a pivotal role in the binding of Gα_q/11_ and transduction of Gα_q/11_-mediated signaling [24]. In Figure 2B, the PAR1^R205A–^Ven mutant failed to stimulate BRET signals in the presence of Gα_11_. As a result, we can confirm that Gα_q/11_ plays a crucial role in the induction of PAR1·RGS2 complex formation. Moreover, our results (Fig. 4) suggest that i3 loops of PAR are necessary for binding to RGS2 and RGS4. Taken together, the i2 and i3 loops of PAR1 mainly contribute to binding to Gα_11_ and RGS2, respectively.

Like RGS2, RGS4 can bind to both G_i_ and G_q_ families and prohibit Gα-related GPCR pathways [11], [17], [18], [37]. Interestingly, we found that only Gα_o_ can stimulate BRET signals between PAR1-Ven and RGS4-Luc (Fig. 3). Although Gα_i_ shares high sequence identity with Gα_o_, we find that Gα_i_ failed to alter BRET signals. Evidence suggests that Gα_o_ has a function distinct from Gα_i_. Gα_o_ binds directly to the catalytic subunit of PKA, and interferes with nuclear translocation of PKA [38]. Activation of Gα_o_, but not Gα_i_, is sufficient to promote neuritogenesis by modulating RapGAP activity in Neuro2A neuroblastoma cells [39], [40]. Therefore, Gα_o_ seems to have functions distinct from Gα_i_. PAR1 can couple to multiple G proteins from different G protein families raising the possibility that other Gα_i_ family members may recruit specific RGS proteins to PAR1 [23]. PAR1 couples most robustly to Gα_o_ and this interaction may selectively recruit RGS4 to PAR1 for distinct functions as well [23].

In our previous study, PAR1-induced MAPK/ERK activation was completely blocked by PTX in COS7 cells [23], indicating that this activity is primarily mediated by the G_i/o_ family. We find that RGS2 and RGS4 each inhibited PAR1-mediated MAPK/ERK signaling pathway (Fig. 5) suggesting that these RGS proteins prohibited PAR1/Gα-mediated MAPK/ERK signaling. In addition, both RGS2 and RGS4 inhibited PAR1/Gα_q/11_-medicated calcium signaling, but not PAR1/Gα_12/13_-mediated Rho signaling. Our BRET studies here indicate that RGS2 and RGS4 bind to Gα_q/11_ and to Gα_o_, respectively, to form functional complexes with PAR1. Taken together, our findings suggest that PAR1/G_q/11_ recruits RGS2 to mediate calcium signaling, and that PAR1/G_i/o_ recruits RGS4 to mediate MAPK/ERK signaling, although we note that RGS2 and RGS4 may also suppress MAPK/ERK signaling calcium signaling, respectively, by binding G proteins directly independent of PAR1.

In summary, we observed the binding properties between PAR1, Gα and RGS proteins, including RGS2 and RGS4 in live cells, and we also tested whether RGS2 and RGS4 inhibit the PAR1/Gα-mediated signaling pathway. RGS2 and RGS4 selectively bind to and inhibit PAR1/G_q/11_- and PAR1/G_i/o_-mediated signals, respectively, and there is a difference in the binding properties of RGS proteins with PAR1 depending on the Gα. Our findings are consistent with a very recent report showing that PAR1 signaling is modulated by R4 family members of RGS proteins that include RGS2 and RGS4 [41], and provide new insights into molecular mechanisms for how GPCR, RGS and Gα form functional preferred signaling complexes within cells. These studies could be of value in developing small molecule modulators of PAR1 signaling pathways.

## Supporting Information

Figure S1 **COS7 cells transfected with an increased amount of PAR1-Ven (0, 0.25, 0.5, 1.0, 1.5, 2.0 µg) together with a fixed amount of RGS2-Luc (35 ng) (Red symbols) or RGS14-Luc (5 ng) (Black symbols) were subjected to the BRET assay in both the absence and presence of 0.5 µg of Gα (Blue symbols) [Gα_i_ (A), Gα_o_ (B), Gα_12_ (C), Gα_13_ (D) and Gα_s_ (E)], and 30 µM of TFLLR.** Net BRET signals are shown between PAR1-Ven and either RGS2-Luc or RGS14-Luc. The black and red data and plots in (B) – (E) were identical to those in (A). Abbreviations used are R14-L  =  RGS14-Luc; R2-L  =  RGS2-Luc.
(TIF)

Figure S2 **COS7 cells were transfected with an increased amount of PAR1-Ven (0, 0.1, 0.25, 0.5, 1.0 and 1.5 µg) together with fixed an amount of RGS4-Luc (45 ng) (Red Symbols) or RGS14-Luc (5 ng) (Black Symbols), and the cells were subjected to the BRET assay in both the absence and presence of 0.5 µg of Gα (Blue symbols) [Gα_i_ (A), Gα_q_ (B), Gα_11_ (C), Gα_12_ (D), Gα_13_ (E), and Gα_s_ (F)] and 30 µM of TFLLR.** Net BRET signals are shown between PAR1-Ven and either RGS4-Luc or RGS14-Luc. The black and red data and plots in (B) – (F) were identical to those in (A). Abbreviations used are R14-L  =  RGS14-Luc; R4-L  =  RGS4-Luc.
(TIF)
