# Supplementary figures and images for: Regulator of G Protein Signaling 2 (RGS2) and RGS4 Form Distinct G Protein-Dependent Complexes with Protease Activated-Receptor 1 (PAR1) in Live Cells
Source: PLoS One. 2014 Apr 17;9(4):e95355. doi: 10.1371/journal.pone.0095355 (PMC3990635; doi:10.1371/journal.pone.0095355)

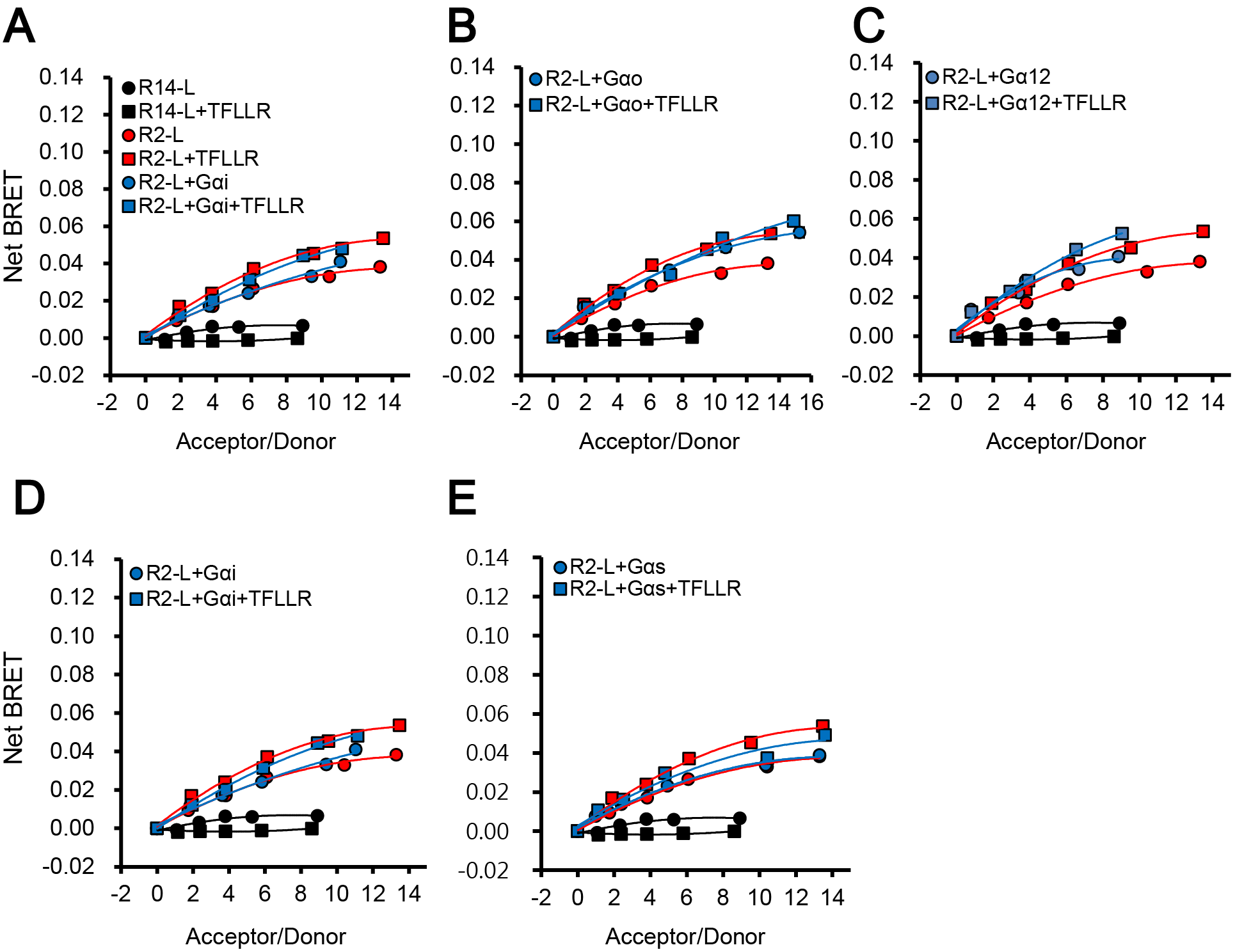

Supplement: Figure S1 — COS7 cells transfected with an increased amount of PAR1-Ven (0, 0.25, 0.5, 1.0, 1.5, 2.0 µg) together with a fixed amount of RGS2-Luc (35 ng) (Red symbols) or RGS14-Luc (5 ng) (Black symbols) were subjected to the BRET assay in both the absence and presence of 0.5 µg of Gα (Blue symbols) [Gαi (A), Gαo (B), Gα12 (C), Gα13 (D) and Gαs (E)], and 30 µM of TFLLR. Net BRET signals are shown between PAR1-Ven and either RGS2-Luc or RGS14-Luc. The black and red data and plots in (B) – (E) were identical to those in (A). Abbreviations used are R14-L = RGS14-Luc; R2-L = RGS2-Luc. (TIF) [file pone.0095355.s001.tif]

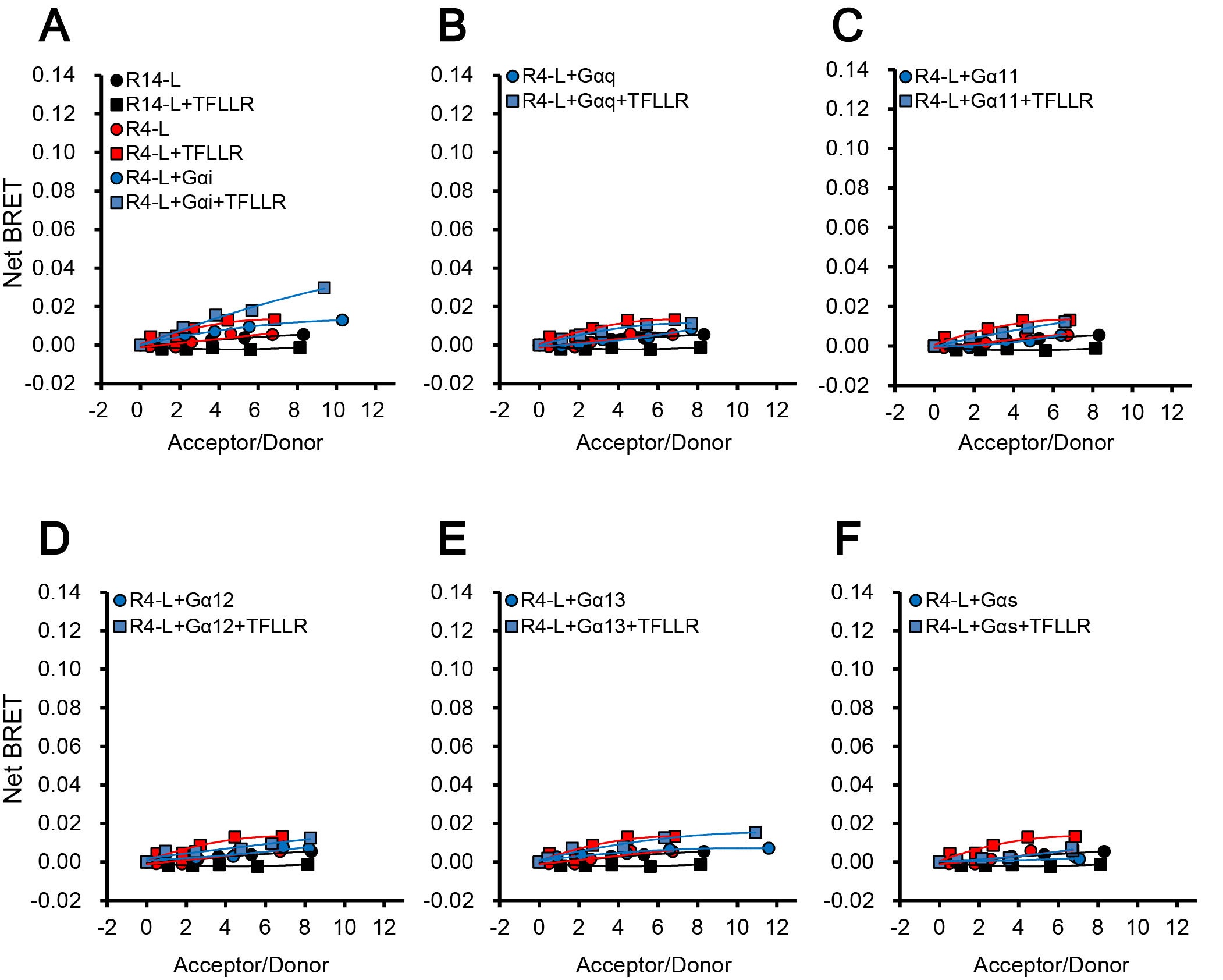

Supplement: Figure S2 — COS7 cells were transfected with an increased amount of PAR1-Ven (0, 0.1, 0.25, 0.5, 1.0 and 1.5 µg) together with fixed an amount of RGS4-Luc (45 ng) (Red Symbols) or RGS14-Luc (5 ng) (Black Symbols), and the cells were subjected to the BRET assay in both the absence and presence of 0.5 µg of Gα (Blue symbols) [Gαi (A), Gαq (B), Gα11 (C), Gα12 (D), Gα13 (E), and Gαs (F)] and 30 µM of TFLLR. Net BRET signals are shown between PAR1-Ven and either RGS4-Luc or RGS14-Luc. The black and red data and plots in (B) – (F) were identical to those in (A). Abbreviations used are R14-L = RGS14-Luc; R4-L = RGS4-Luc. (TIF) [file pone.0095355.s002.tif]
